# BENEFIT for all: An ecosystem to facilitate sustained healthy living and reduce the burden of cardiovascular disease

**DOI:** 10.1177/2047487318816388

**Published:** 2018-11-28

**Authors:** Mike Keesman, Veronica Janssen, Hareld Kemps, Monika Hollander, Wilma Scholte op Reimer, Lisette van Gemert-Pijnen, Arno Hoes, Wessel Kraaij, Niels Chavannes, Douwe Atsma, Roderik Kraaijenhagen, Andrea Evers, on behalf of the BENEFIT consortium

**Affiliations:** 1 Health, Medical, and Neuropsychology Unit, Leiden University, The Netherlands; 2 Department of Cardiology, Leiden University Medical Center, The Netherlands; 3 Department of Cardiology, Máxima Medical Center Veldhoven, The Netherlands; 4 Julius Centre for Health Sciences and Primary Care, University Medical Centre Utrecht, The Netherlands; 5 Department of Cardiology, Academic Medical Center, The Netherlands; 6 Faculty of Health, Amsterdam University of Applied Sciences, The Netherlands; 7 Centre for eHealth and Wellbeing Research, University of Twente, The Netherlands; 8 Leiden Institute for Advanced Computer Science, Leiden University, The Netherlands; 9 Department of Public Health and Primary Care, Leiden University Medical Center, The Netherlands; 10 NDDO Institute for Prevention and Early Diagnostics (NIPED), The Netherlands; 11 Vital10, The Netherlands; 12 Department of Psychiatry, Leiden University Medical Centre, The Netherlands

A healthy lifestyle forms the basis for preventing cardiovascular disease (CVD).^1^ However, initiating and maintaining a healthy lifestyle is notoriously difficult. Despite large investments in cardiac prevention and rehabilitation programmes, the majority of people with CVD still do not achieve guideline treatment goals for cardiovascular risk management, such as lipid targets or receiving lifestyle modification programmes.^2^ The following pillars, each from a different discipline, are known as instrumental to facilitate sustained healthy living: (a) target both individual and environmental lifestyle factors (social and behavioural sciences;^3^ (b) develop interventions in continuous co-creation with stakeholders (design sciences);^4^ (c) ensure continuous, transmural access to these interventions (medicine, data and implementation science;^5^ and (d) create public–private partnership (economics, management science).^6^ An ecosystem for healthy living linking each of these pillars is currently being designed, implemented, and evaluated nationwide in The Netherlands for people with or at high risk of CVD.

## Target both individual and environmental lifestyle factors

Following the notion that people act in a reasoned fashion, many rehabilitation and lifestyle modification programmes focus on enhancing an individual’s health literacy, efficacy beliefs and the motivation to adopt a healthy lifestyle. While effective,^7^ complimentary approaches are required, as strong drivers of health behaviours are often non-reasoned,^3^ such as habits, hormones and the desire for short-term rewards. Programmes that modulate unreasoned processes to favour the healthy option or that enhance skills, such as progress monitoring and action planning, empower people to live healthily despite these unreasoned processes.^8^

Even when motivated and skilled to live healthily, the modern-day ‘obesogenic’ environment often triggers the unreasoned processes that make people engage in unhealthy behaviours. When hungry after a long day of work and walking past an inexpensive fast-food restaurant, for instance, it is easy and attractive to eat an unhealthy snack. Such unhealthy choices are preventable by modulating environmental factors to make it easy and attractive to live healthily instead.^3^ Placing fruits instead of unhealthy snacks at cash registers and using technology automatically to dim lights around the intended bedtime, makes it easier to live healthily, i.e. nudging. Providing material rewards contingent on healthy choices, or engaging in challenges makes it more attractive to live healthily. Meta-analyses indeed show that this environmental approach increases the adoption of a healthy lifestyle.^9^ Crucially, interventions need to target individual and environmental factors simultaneously to facilitate sustained healthy living.^3^

## Develop interventions in continuous co-creation with stakeholders

In intervention development, content is usually developed first, and after that technology and context of use comes to mind. Yet, while technologies provide opportunities for multi-party interaction and collaborative working with patients, they require significant tailoring to the individual and the context of use. Long-term uptake and impact of evidence-based interventions are dependent on the needs, context and technology for implementation in day-to-day routines of healthcare, living and working.^4^ For instance, if patients or healthcare professionals do not possess the required time or skills to use an intervention, its working mechanisms are severely limited, reducing its potential impact. To ensure long-term uptake of interventions, the design of technology-mediated interventions requires participatory development from ideation to roll-out. All stakeholders from the affected ecosystem need to be continuously involved, from patients and healthcare professionals, to technology developers and policy makers. This process of stakeholder involvement during development, evaluation and implementation, is outlined in the widely used CeHReS roadmap for participatory development.^4^ Such co-creation is necessary to ensure sustained uptake and impact of the intervention on health and wellbeing, and to promote an efficient organisation of healthcare.^4^

## Ensure continuous transmural access to interventions

When there is a gap in access to individual or environmental-level interventions, such as when transitioning levels of care between healthcare professionals or when cardiac rehabilitation ends, people often relapse into their previous unhealthy behavioural patterns.^9^ To achieve sustained healthy living, transmural access to lifestyle interventions and data is important, e.g. by integrating various eHealth technologies.^10^ A prime example is a personal digital healthcare environment that stores a patient’s health-related information, connects with wearables, provides access to lifestyle interventions and supports tele-consultation. Adequate data governance, ensuring flexible yet safe data infrastructure, is an important pre-condition for such an environment. A patient can for instance decide to share these data with health professionals in the different care settings, or with peers, to involve them. This enables the use of goals and data from cardiac rehabilitation to cardiometabolic risk management in hospital and primary care setting, and continuation of interventions in the home situation. In addition, by enabling digital coaching and monitoring of patients, this can continue without physical presence. This added flexibility facilitates coaching in the daily environment of patients, and real time feedback increases adherence to lifestyle interventions. For health professionals it enables long-term coaching, and for patients it facilitates self-management of healthy living.

## Public–private partnership

The long-term implementation of comprehensive interventions such as a personal digital healthcare environment cannot be achieved by one party alone.^6^ For one, continuously engaging in a healthy lifestyle requires that interventions are embedded in day-to-day routines of care in public settings, but also in day-to-day life in private settings. In addition to the efforts of health practitioners, private parties can encourage and seduce patients to make healthy choices in daily life by nudging strategies, marketing, discounts and loyalty programmes. In addition, private investment complements public funding in terms of capital and expertise, such as in operational efficiency and sustainable business models. Private parties such as insurance companies also have financial incentives to strive for a reduction in CVD. Furthermore, while private parties have the means to mass-produce health applications and devices, much know-how is developed through publicly funded scientific research.^6^ Public–private partnership hereby safeguards the social responsibility of all parties involved.^6^ Altogether, public–private partnership in CVD management will enable an ecosystem that promotes sustained healthy living to the benefit for all.

## Realising an ecosystem approach

An ecosystem linking the necessary pillars to facilitate sustained healthy living is currently taking shape through the BENEFIT consortium in The Netherlands, a public–private partnership uniting academic centres, hospitals, rehabilitation centres, general practices, companies and patient federations. A patient enrolled in cardiac rehabilitation receives, in addition to usual care, access to the BENEFIT environment and loyalty programme supported by a digital personal health application (PHA). This PHA connects interventions from various private and public parties, such as online or offline coaching, lifestyle modification applications, and self-monitoring devices. Healthy living is made more attractive with challenges and rewards for attending appointments with healthcare providers, adhering to evidence-based lifestyle change programmes and coaching, and self-monitoring lifestyle behaviours. These rewards can be exchanged for discounts on health-related goods and services. This increases business for these organisations, creating an incentive for offering healthy rather than unhealthy products. After rehabilitation ends, patients continue using the PHA, ensuring continuity of care and transmural access to their data and lifestyle interventions. The BENEFIT ecosystem is continuously improved through participatory development and co-creation with the relevant stakeholders, such as CVD patients and health professionals. Rigorous scientific evaluation with a stepped-wedge roll-out in hospitals, general practices and municipalities will ultimately determine the added value of the BENEFIT ecosystem for increasing sustained healthy living and reducing CVD.

## Conclusion

A public–private ecosystem for sustained healthy living empowers people with or at high risk of CVD to adhere to guideline standards for healthy living despite living in an ‘obesogenic’ environment, subsequently reducing CVD risk factors and medication requirements. Health professionals will spend less time on obtaining routine measurements and referrals to lifestyle interventions, and can provide better healthcare, facilitated by the PHA. Private parties will have increased health-related revenue. Overall, an ecosystem approach for healthy living has the potential to engage public as well as private parties to decrease the burden of CVD, to bring benefit for all.
